# Psychosocial and relational resilience during the COVID-19 pandemic: Evaluating sexual health and coping strategies in intimate relationships

**DOI:** 10.1192/j.eurpsy.2025.2308

**Published:** 2025-08-26

**Authors:** S. Irnat, H. Harifi, F. Hadrya, O. Erefai, A. Soulaymani, M. Anssoufouddine, M. Abdalli Mari, H. Hami

**Affiliations:** 1 Laboratory of Biology and Health, Faculty of Science, Ibn Tofail University, Kenitra; 2 University Hassan First of Settat, Higher Institute of Health Sciences, Health Sciences and Technologies Laboratory, Settat; 3 Higher Institute of Nursing Professions and Health Techniques, Rabat, Morocco; 4 Medical Service, Regional Hospital Center of Anjouan, Mutsamudu, Comoros

## Abstract

**Introduction:**

The COVID-19 pandemic has significantly altered daily life and affected the sexual dynamics of couples across various contexts. Enforced lockdowns and pervasive social isolation, coupled with heightened anxiety associated with the pandemic, have profoundly affected mental health and intimate relationships, affecting sexual satisfaction among couples. This narrative review examines the psychosocial effects of these changes on couples’ sexuality, focusing on the challenges and coping strategies adopted by couples to mitigate the adverse effects and enhance intimate relationship quality.

**Objectives:**

This review systematically analyzes the psychosocial impacts of the pandemic on couples’ sexuality, using post-2020 literature to better understand these dynamics and enhance psychosexual support.

**Methods:**

An extensive literature review was conducted across PubMed, Scopus, and Google Scholar, focusing on keywords such as “COVID-19,” “sexual health,” “intimacy,” “divorce,” and “coping strategies.” Ten high-quality studies published after 2020 were selected based on their methodological rigor and relevance to couples’ dynamics. These studies include a mix of quantitative and qualitative research and systematic reviews, providing a broad yet detailed perspective on the topic.

**Results:**

The findings reveal that the pandemic has significantly diminished couples’ sexual desire and relationship satisfaction. Notable psychosocial effects include increased anxiety, stress, depression, and relational conflicts. Modifications in sexual frequency and quality were noted, yet many couples have adopted effective coping strategies such as enhanced communication, couples therapy, shared stress management activities, and the utilization of online psychological support resources, which have helped strengthen relationships during the pandemic.

**Conclusions:**

The pandemic has profoundly influenced couples’ sexual and relational health, introducing psychosocial stressors. Despite these challenges, the resilience displayed by couples through diverse coping strategies highlights their adaptive capacity. This review emphasizes the need for mental health professionals to integrate targeted interventions to support couples’ well-being and prepare them for potential future crises.

**Disclosure of Interest:**

None Declared

